# Capacity building among frontline health workers (FHWs) in screening for cardiovascular diseases (CVDs): Findings of an implementation study from Bihar, India

**DOI:** 10.3934/publichealth.2023017

**Published:** 2023-03-30

**Authors:** Neeraj Agarwal, CM Singh, Bijaya Nanda Naik, Abhisek Mishra, Shamshad Ahmad, Pallavi Lohani, Saket Shekhar, Bijit Biswas

**Affiliations:** 1 Community & Family Medicine, AIIMS, Bibinagar, Telangana, India; 2 Community & Family Medicine, AIIMS, Patna, Bihar, India; 3 Community Medicine & Family Medicine, AIIMS, Bhubaneswar, Odisha, India; 4 Community Medicine, Madhubani Medical College & Hospital, Keshopur, Bihar, India; 5 Community Medicine, Rama Medical College Hospital and Research Centre, Kanpur, U.P., India; 6 Community & Family Medicine, AIIMS, Deoghar, Jharkhand, India

**Keywords:** capacity building, mass screening, noncommunicable diseases, cardiovascular diseases, community health workers, India

## Abstract

**Background:**

Community-based screening is one of the key preventive strategies to tackle the ever-rising burden of non-communicable diseases (NCDs) under the National Programme for Prevention & Control of Cancer, Diabetes, Cardiovascular Diseases and Stroke (NPCDCS).

**Objective:**

The current study was aimed to build capacity among frontline health workers (FHWs) in screening for cardiovascular diseases (CVDs) under NPCDCS in the selected districts of Bihar state.

**Methodology:**

This was an implementation study with follow-up components, conducted among 75 FHWs [14 auxiliary nurse midwives (ANMs) and 61 accredited social health activists (ASHAs)] from 15 primary healthcare facilities across four districts of Bihar state from October 2019 to September 2021. The selected FHWs were initially trained on NPCDCS for a day, including pre- and post-training knowledge assessment. Then, supportive supervision (SS) visits using a predesigned questionnaire were done.

**Results:**

The pre- and post-training mean knowledge scores of the FHWs were 12.9 and 22.1, respectively, with an overall effect size of 2.5. During SS visits, only 20.0% of the visited primary healthcare facilities had all the required logistics to conduct weekly NCD screening clinics for CVDs. Considering different measurements and operative skill proficiencies of FHWs, waist circumference skills (41.7% for ANMs and 50.8% for ASHAs), followed by blood pressure (BP) (41.7%) and random blood sugar (RBS) measurement (25.0%), were found to be the most deficient skills (among ANMs). Moreover, the quality of initial and follow-up home visits was found to be satisfactory for only 54.1% of the ASHAs. The reported barriers of NCD screening were reported to be non-cooperation, unawareness among community dwellers, lack of knowledge and skill of FHWs, logistic constraints and delayed honorarium credit.

**Conclusion:**

One-day training on NCDs for FHWs was quite effective. However, for translating all the desired skills for CVD screening into action, periodic training needs assessment, and SS of FHWs might be fruitful.

## 1. Introduction

Non-communicable diseases (NCDs) are accountable for one sixth of the disability adjusted life years (DALYs) in India [1]. Each year nearly six million people succumbed to NCDs (cancer, stroke, cardio-respiratory diseases and diabetes) in the country. Invariably every fourth Indian had potential risk of dying due to NCDs before they attain their seventh decade of life [2]. The prevalence of communicable (42.6%) and non-communicable (47.6%) diseases are quite high in the state of Bihar. In recent times, a gradual epidemiological transition from communicable to non-communicable disease burden in the state has also been documented [3]. As per NCD Rural India (NCDRI) study, the prevalence of hypertension in rural Bihar is 27.3% in women and 27.6% in men aged 35–70 years, and these values are approximately three-times higher in women and over two-times higher in men compared to the recent National Family Health Survey 5 (NFHS-5) report [4],[5].

To prevent and control the rising burden of NCDs in India, the National Programme for Prevention & Control of Cancer, Diabetes, Cardiovascular Diseases and Stroke (NPCDCS) was initiated in the year 2010. As part of this program, community dwelling adults aged ≥30 years are being screened for common NCDs (i.e., diabetes, hypertension and common cancers [oral, breast and cervix]) by frontline health workers (FHWs). The risk ascertainments of the individual adults in the community were done using the Community Based Assessment Checklist (CBAC), which assesses an individual for six risk factors, two non-modifiable (age, family history of NCD) and four modifiable (tobacco consumption, alcohol consumption, physical activity level and waist circumference [WC]). Individuals with higher CBAC scores (≥4) are being prioritized and mobilized by accredited social health activists (ASHAs) to attend weekly NCD clinics where auxiliary nurse midwives (ANMs) reverify CBAC entries and measure blood pressure (BP) and random blood sugar (RBS) of individuals. Then, based on the screening results, the ANM either advises lifestyle modification or refers the case to the medical officer (MO) for further management [6],[7].

FHWs (ANMs and ASHAs) for conducting CVD screening are expected to be adequately knowledgeable and skilled. Additionally, FHWs are expected to counsel a diagnosed NCD patient regarding the importance of adoption of a healthy lifestyle, medication adherence and doctor consultation [6]–[8]. Despite this, variable knowledge (41.2–100.0%) and skill (23.5–94.1%) related to NCDs among FHWs has been documented by prior studies in this regard [9],[10]. Moreover, what was more concerning is that only 1.1% of rural and 2.3% of urban public health facilities of NPCDCS implementing districts reported having adequate infrastructure and logistic supply to provide NCD care [11]. High burdens of NCDs and their risk factors in the state of Bihar demonstrated need for effective implementation of NPCDCS in the state. However, programmatic barriers, adequate technology and trained manpower availability in the primary healthcare facilities of the state are largely unexplored. In the backdrop of this, the current operational research was formulated to build capacity among FHWs in screening for CVDs under NPCDCS in the selected districts of Bihar state. Simultaneously, the study tried to elicit implementation challenges for CVD screening. Additionally, the study also has elicited effects of a one-day, participatory, structured training program on NCDs on knowledge levels of the FHWs of the study area. The study will provide much needed feedback to the policymakers of the program to enunciate feasible course corrections for its effective implementation. The study also concentrated on skill development of FHWs, which will serve to raise the standard of healthcare in the study area.

## 2. Materials and methods

**Figure 1.**
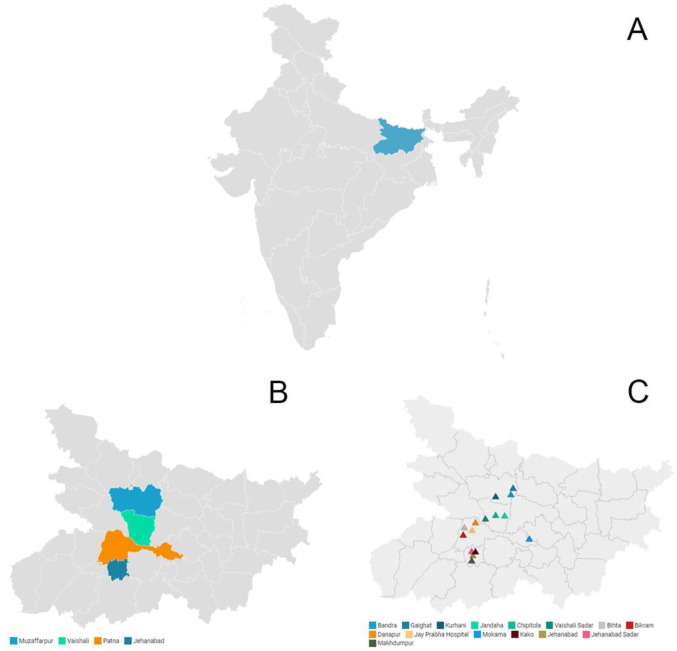
Map of India and Bihar showing the study area: A: Map of India showing the study state Bihar; B: Map of Bihar showing the study districts; C: Map of Bihar showing the study primary healthcare facilities.

**Figure 2.**
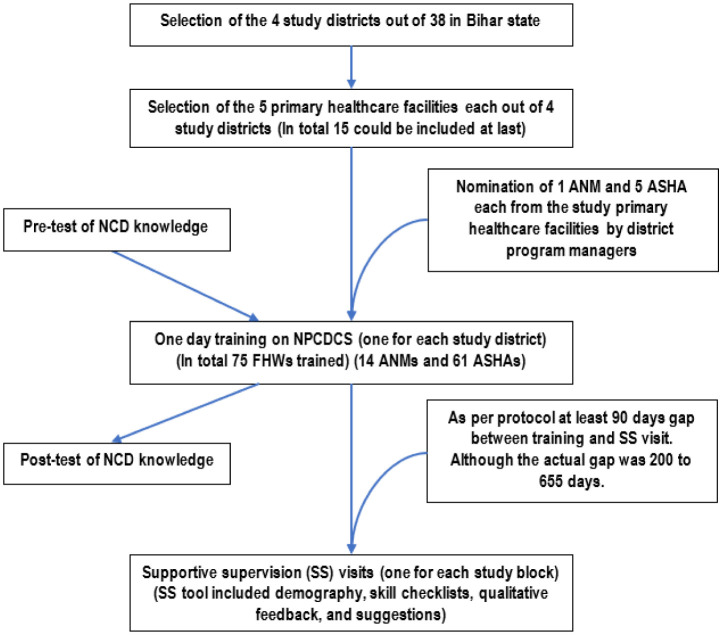
Flowchart showing methodology of the study. NPCDCS: National Programme for Prevention & Control of Cancer, Diabetes, Cardiovascular Diseases and Stroke; NCD: non-communicable disease; ANM: auxiliary nurse midwife; ASHA: accredited social health activist; FHW: frontline health worker.

This was implementation research with follow up components, conducted among 75 selected FHWs from 15 primary healthcare facilities across four districts of Bihar state (namely, Patna, Jehanabad, Muzaffarpur and Vaishali) from October 2019 to September 2021. The 4 study districts from the total sampling frame of 38 districts were chosen using convenience sampling based on their vicinity to Patna, feasibility and operational constraints. This was followed by nomination of 5 primary healthcare facilities from each of the study districts by program administrators of that specific district. However, considering logistic and feasibility issues, a total of 15 primary healthcare facilities could be included in the study (5 from Patna, 4 from Jehanabad, 3 each from Muzaffarpur and Vaishali) (Figure 1). The project comprised two main phases: Phase I, training of the FHWs on NPCDCS, and Phase II, supportive supervision (SS) for quality improvement (QI) of CVD screening in the study area (Figure 2).

### 2.1. Phase I: Training of the FHWs on NPCDCS

Program administrators of the selected districts were contacted at least one month before the planned training to nominate one ANM and five ASHAs from the selected primary healthcare facilities for undergoing training on NPCDCS. Additionally, one medical officer (MO) and lab technician (LT) from the study health facilities were also invited to be sensitized regarding the program. Considering feasibility, available logistics and contents of the training, it was agreed to be of one day duration after consultation with the concerned program managers. For each study district, the organized training contained the following sessions: epidemiology of CVD and public health importance of screening; stakeholder's responsibility; demonstration & hands on: CBAC form and family folder; overview and hands on: NCD portal and demonstration & hands on: anthropometry, BP measurement, RBS estimation. Each session was facilitated by either a faculty or senior resident (SR) of the Community & Family Medicine (CFM) department of AIIMS Patna, excepting the session on the NCD portal, which was taken by a nominated resource person from the Tata Institute of Social Sciences (TISS). Notably, the NCD portal is software developed by the Ministry of Health and Family Welfare (MOHFW), Government of India (GOI), for management of population-based screening of common NCDs to prevent, screen and control these diseases. For ASHA/ANM, it has a tablet or mobile application, and for healthcare facilities, it has a web-based interface. Some typical features of this software are estimation of population to be screened for NCDs, creation and retrieval of individual health records, auto generation of screening due list with alert, report generation for a geographical area, referral linkages, etc.

The training session began with formal introduction of the trainers and trainees, followed by self-administration of pre-test knowledge questionnaire. After completion of the pre-test knowledge questionnaire, the training kit (containing a file folder, writing pad, pen, measuring tape, NPCDCS module developed for the project and a set of CBACs with family folder) was provided to each trainee. The NPCDCS module was developed especially for this project. It served mainly two uses: First, it reinforced must-know attributes of NPCDCS to the trainees; second, it also served as a counseling aid for ASHAs and ANMs while conducting NCD screening and referral related activities. The module comprised modifiable and non-modifiable risk factors, high risk population, early signs, symptoms and complications of CVDs; steps of WC measurement; calculation and interpretation of body mass index (BMI); early signs and symptoms of common cancers; healthy lifestyle including balanced diet; screening and referral algorithm; and specific role of ASHAs and ANMs in the Program [6],[7],[12]. All the sessions were participatory in nature. At the end of each session, questions were asked to check the participants' understanding. In case of any discrepancy, the concerned portion of the session was repeated. After completion of the scheduled training sessions, the post-test questionnaire was self-administered among the participants. In total, 75 FHWs (14 ANMs and 61 ASHAs) were trained in four distinct training sessions for the study districts.

There were in total 28 items in the pre- and post-test knowledge questionnaires. It contained items on risk and protective factors of common NCDs; target population, frequency and forms to be filled for NCD screening; role and responsibilities of each stakeholder; and interpretation of BP and RBS readings for ascertainment of referral need. All these items were selected and incorporated after thorough literature search and in consultation with the experts in the field [6]–[8]. The NCD knowledge items had shown acceptable internal consistency (Cronbach's alpha: 0.735). For each correct response, the study participant received 1 point, except for item 6b, for which a correct response received 2 points. The minimum and maximum attainable scores were 0 and 29, respectively. The minimum and maximum attained pre-test scores were 8 and 18, respectively, whereas for the post-test they were 15 and 29, respectively.

### 2.2. Phase II: SS for QI of CVD screening in the study area

This was initiated only after three months of training of FHWs of a study district. The SS visit was done by a faculty member or SR of CFM department of AIIMS Patna with an accompanying junior resident. Before rendering SS visit to a specific primary health care facility of a district, program administrators of that district were contacted well in advance for facilitation of mobilization of FHWs trained under the project to the concerned facility. The gap between training and SS visit varied between 200 and 655 days, with a median (interquartile range [IQR]) of 490 (213–641) days. The ANMs who were notified for SS visits were asked to carry their tablet with NCD portal preinstalled. Similarly, ASHAs were asked to carry a filled CBAC and family folder to elicit administration difficulty if any. During SS visits, the data was collected with the help of a predesigned SS tool.

The SS tool for the study was developed after thorough literature search and in consultation with the experts in the field [6]–[8],[13]–[16]. It comprised background details of the primary healthcare facility visited (name, type, location, population aged ≥30 years, existing manpower). This was followed by an individual checklist for ANMs and ASHAs. The ANM checklist comprised her age, qualification, work experience, logistics availability for CVD screening, checklist for NCD portal skills, weight, height, WC, BP and RBS measurement. The SS checklist of ASHAs comprised name of ASHA, age, qualification, work experience, supervisor details, checklist of logistics availability, 1^st^ and follow-up home visit and WC measurement. Different practical skills of FHWs were evaluated with the help of available volunteers (i.e., patients or other health workers). The quality of each measurement was rated as excellent (having >80% skill), satisfactory (having 50–80% skill) or poor (having <50% skill). On conclusion of each FHW evaluation, they were asked about existing challenges faced when delivering CVD screening and the suggested ways to overcome those. In total, 14 ANMs and 61 ASHAs could be interviewed during SS visit. After eliciting the skill level of FHWs of each facility, the visiting SS experts tried to plug the knowledge and skill gap among study FHWs. This was done en masse by demonstration, explanation of important concepts and answering existing queries.

### 2.3. Ethics approval of research

The research was approved by the institutional ethics committee (IEC) of AIIMS Patna (Approval. No: AIIMS/Pat/IEC/2019/401). Informed written consent of the study participants was taken before their inclusion.

### 2.4. Statistical analysis plan

The information collected during the research was digitalized using Microsoft Excel and later analyzed using the Statistical Package for the Social Sciences (SPSS) (Version 22). Qualitative variables were reported using frequency (percentage), whereas mean (standard deviation [SD]) or median (IQR) was used to report quantitative variables based on normality of the data. The normality of the knowledge score was ascertained via q-q plot. To measure the effect size of the training, paired ‘t’ test was used, and the effect size was expressed in terms of Cohen's D. For all quantitative analysis statistical significance was set at a p value < 0.05. Meanwhile, for the qualitative responses, inductive and deductive coding was done to derive themes of barriers and suggested solutions for effective implementation.

## 3. Results

The mean age of the study FHWs was 41.4 years (range: 28–57 years). Three fifths (61.4%) of the FHWs were educated up to tenth grade, with mean years of schooling of 10.8 years (range: 0–15 years). Four out of every five FHWs surveyed (82.7%) had work experience of more than five years, with mean years of experience of 12.3 years (range: 2–34 years). The pre- and post-training mean NCD related knowledge scores of the FHWs were 12.9 and 22.1, respectively, with an overall effect size of 2.5. The effect size for training was 1.3 times more for ANMs (3.1) compared to the ASHAs (2.4) (Table 1).

**Table 1.** Effect of Training on NCD related knowledge among FHWs.

	Variable	Mean ± SD	Mean Difference ± SD	Effect Size*	p-value
Overall (N = 75)	Pre-Test Knowledge Score	12.9 ± 2.3	9.1 ± 3.7	2.5	<0.001^#^
	Post-Test Knowledge Score	22.1 ± 3.6			
ANMs (N = 14)	Pre-Test Knowledge Score	14.1 ± 2.5	11.3 ± 3.7	3.1	<0.001^#^
	Post-Test Knowledge Score	25.5 ± 1.9			
ASHAs (N = 61)	Pre-Test Knowledge Score	12.7 ± 2.2	8.6 ± 3.6	2.4	<0.001^#^
	Post-Test Knowledge Score	21.3 ± 3.4			

*Note: *Cohen's d, ^#^Paired t test; ANM: auxiliary nurse midwife; ASHA: accredited social health activist; FHW: frontline health worker; NCD: non-communicable disease; SD: standard deviation.

**Figure 3.**
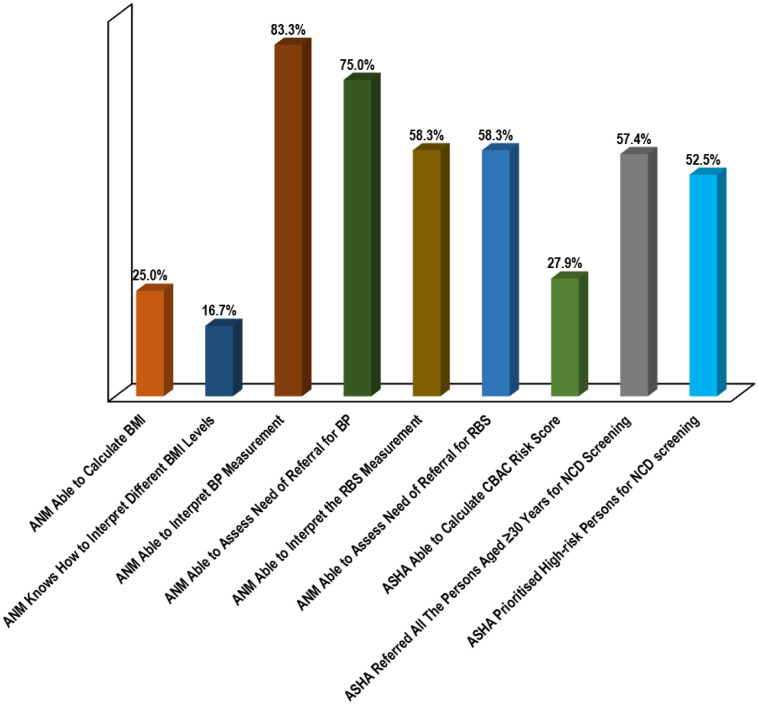
Bar chart showing level of skills regarding different attributes of CVD screening of the surveyed FHWs during the SS visits. ASHAs, N = 61, and for ANMs, N = 14; ANM: auxiliary nurse midwife; ASHA: accredited social health activist; BMI: body mass index; BP: blood pressure; CBAC: community-based assessment checklist; CVD: cardiovascular disease; FHW: frontline health worker; NCD: non-communicable disease; RBS: random blood sugar; SS: supportive supervision.

**Figure 4.**
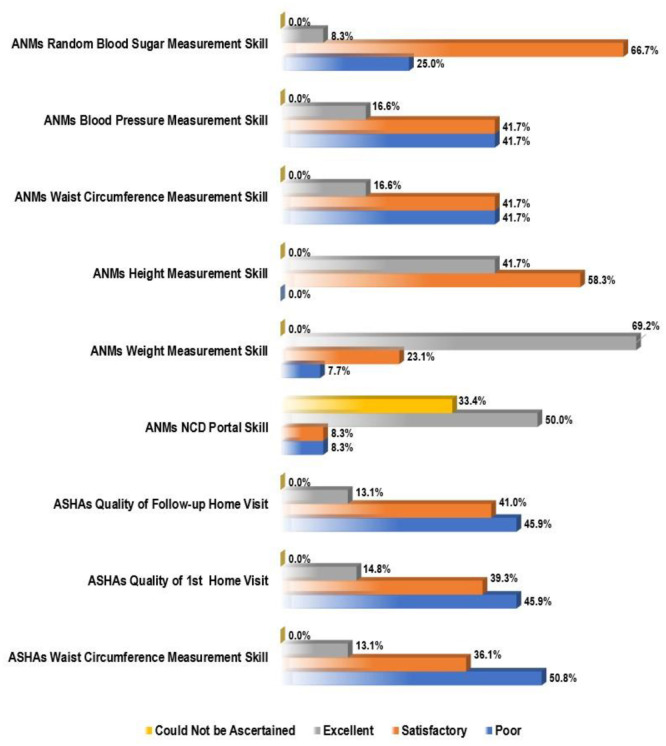
Bar chart showing levels of skills regarding different attributes of CVD screening of the surveyed FHWs during the SS visits. For ASHAs, N = 61, and for ANMs, N = 14; ANM: auxiliary nurse midwife; ASHA: accredited social health activist; CVD: cardiovascular disease; FHW: frontline health worker; NCD: non-communicable disease; SS: supportive supervision.

Overall, only 20.0% of the primary healthcare facilities had all the required logistics to conduct a weekly NCD screening clinic for CVDs. The most deficient logistical feature was observed to be internet connection for syncing NCD data (67.7%), followed by functional tablet with preinstalled NCD portal (33.3%), glucometer strips (25.0%), stadiometer (25.0%), non-stretchable measuring tape (16.7%). Meanwhile, 8.3% of primary healthcare facilities lacked a weighing machine, BP machine, glucometer, lancet, cotton and gloves or handwashing facility. Considering logistical supply for enumeration of NCD risks of community dwellers, the family folder was found to be the most deficient logistical feature (50.8%), followed by CBAC form (49.2%), non-stretchable measuring tape (27.9%) and job aids for counseling (24.6%). The most deficient measurement skill was observed to be WC (41.7% for ANMs, 50.8% for ASHAs), followed by BP (41.7%) and RBS (25.0%) (among ANMs). Notably, NCD portal skill of 33.3% of the ANMs could not be ascertained due to non-availability of functional tablet. Considering different calculation and interpretation skills, ANMs were found to be most deficient in interpretation of BMI (83.3%), followed by its calculation (75.0%) and interpretation of RBS reading (41.7%), while for ASHAs it was observed to be calculation of CBAC risk score (72.1%). Quality of initial and follow-up home visits was found to be satisfactory for only 54.1% of the ASHAs (Figures 3 and 4).

The reported barriers of NCD screening were non-cooperation, unawareness among community dwellers; lack of knowledge and skill of FHWs; logistic constraints; and delayed honorarium credit. The qualitative responses of the study participants indicating implementation challenges and their suggested solutions are reported in Table 2.

**Table 2.** Qualitative responses of the study participants indicating barriers in implementation of community-based CVD screening under NPCDCS and their suggested solutions.

Theme	Supporting Verbatim	Solution Suggested
Lack of manpower for NCD screening	-One ASHA facilitator told “For my primary healthcare facility no ANM attended the training. Doing entry in NCD portal, BP and RBS measurement is not my job” -As per seven ASHA “As we were mobilised for COVID-19 and routine immunisation. We could not render enough time for NCD screening”	-As per one ANM “There should be one extra ANM to do NCD related activities as it is very difficult for one ANM to do all these” -As per one ANM “Those who receive NCD related training should be involved in NCD related activities only”
Lengthy tool	-Five ASHA told that “I feel that the forms are too lengthy, community dwellers don't allow us to ask so many questions as they are normally busy” -As per eleven ASHA “Family easily gets irritated due to length of the form”	-As per two ASHAs “Monetary Honorarium for the study participants may increase co-operation” -One ASHA opined that “If we have Good Communication skills may help in overcoming challenges related to NCD screening”
Deficiency in skill and knowledge of the FHWs	-Five ASHA told, “It is very difficult to remember the things what were taught to us”	-Two ANM opined that “We need more training to do NCD Screening” -Three ASHA opined that “We need more training on diet plans.”
Unawareness regarding NCDs in community dwellers	-Five ANM told “non-co-operation of people is the major challenge in NCD screening, when we go to community, they ask that what benefit they will get out of this.”	-Five ANM opined that “Spreading more awareness regarding NCDs might help in increment of NCD screening”
Lack co-operation of the community dwellers	-As per Nine ASHAs “Non-co-operation of family is a big challenge”	-As per three ASHAs “There should be incentives for community dwellers for undergoing NCD screening”
Gender barrier in measurement of WC of males	-As per five ASHA “We feel uncomfortable while measuring WC of males” -As per one ASHA “I feel scared to measure WC of a male, in case he harms me”. -As per one ASHA “We feel uncomfortable while measuring WC of males”	-As per one ASHA “There should be someone (preferably male) who would accompany us to take the measurement of males, create awareness regarding NCDs otherwise this work should be given to someone else” -As per one ASHA “Male health worker should be posted to screen male community dwellers”
Availability of the logistics for NCD screening	-One ANM told “Although were given tablets to enter NCD related data there is no provision for internet connection for syncing the data” -Fifteen ASHA opined that “We were told that we will get ten rupees to fill each CBAC form but normally we are only given one form and asked to get it xeroxed. Due to scarcity of forms sometimes we need to fill details of two individuals / families in one form. We cannot continue screening with money invested from our own pocket.”	-As per two ANM “There should be provision of money for internet data package so that they could sync the data entered in the NCD portal”. -As per eleven ASHAs “Sufficient number of CBAC forms and family folders should be supplied so that NCD related screening could be done.”
Impact of COVID-19 on NCD related services	-One ANM opined “Till date we have screened 1391 people. But due to Second wave of COVID-19 NCD screening stopped. Prior to that We were screening ten persons on average per week” -As per one ASHA “COVID-19 stopped NCD related activities. Prior to that we were doing this”	-Five ANM opined “There should be a separate ANM for discharging routine immunisation and NPCDCS related work as we are very much engaged in COVID-19 vaccination” -As per one ASHA “Soon after the pandemic is over, we will start NCD screening”
Late credit of honorarium	-As per eleven ASHAs “Honorarium for the filled forms is not given yet”	-All the ASHAs suggested “The honorarium must be timely credited”

*Note: ANM: auxiliary nurse midwife; ASHA: accredited social health activist; CBAC: community-based assessment checklist; COVID: coronavirus disease; CVD: cardiovascular disease; FHW: frontline health worker; NCD: non-communicable disease; NPCDCS: National Programme for Prevention & Control of Cancer, Diabetes, Cardiovascular Diseases, and Stroke; RBS: random blood sugar; SS: supportive supervision; WC: waist circumference.

## 4. Discussion

This implementation research with follow up components was primarily aimed to build capacity among FHWs in screening for CVDs in the selected districts of Bihar state. The pre- and post-training mean NCD related knowledge scores of the FHWs were 12.9 and 22.1, respectively, with an overall effect size of 2.5. Community healthcare workers of low- and middle-income countries (LMICs) could be effectively trained for CVD management as reported by a systematic review by Abdel-All et al. [17]. The impact of these training courses depended on the method used for training (lecture/demonstration/hands on), educational level of the trainees, settings of training, etc. The use of audio-visual aids, checking of understanding of the trainees at the end of each session, adoption of participatory problem-solving approach and hands-on exposure might have been fruitful, considering the effect size we could be able to achieve in our study.

In the present study, only 20.0% of the primary healthcare facilities had all the required logistics to conduct weekly NCD screening clinic for CVDs. A study by Krishnan et al. [11] based on results of a National NCD Monitoring Survey (NNMS) stated that in NPCDCS implementing districts of the country, only 1.1% of rural and 2.3% of urban public health facilities had adequate technologies and medicines to provide NCD care. These figures were really concerning. Thus, sufficient fund allocation and supply chain management should be strengthened to ensure availability of logistics to deliver optimal NCD care.

In the present study, 92.3%, 100.0% and 58.3% of the ANMs showed satisfactory to excellent proficiency in weight, height and WC measurement, respectively, which were more compared to the observations of Nebhinani et al. [9], where 64.7%, 58.3% and 5.9% of the front-line workers (FLWs) demonstrated adequate skills for the same, respectively. We observed that 75% of the ANMs of our study demonstrated satisfactory to excellent proficiency in RBS measurement, which was in line with the observations of Nebhinani et al. [9] (76.5%). Concerning BP measurement, the skill level of ANMs (58.3%) in the present study was quite less compared to Nebhinani et al. [9] (76.5%). The variability of observations might have been incurred due to differences in study settings, study subjects, checklist used for ascertainment of skill adequacy, etc.

Lack of manpower for NCD screening emerged as a substantial barrier in implementation. A study from Chandigarh by Monika et al. [18] reported that two thirds of the daily workload of an ANM was devoted for maternal and child health (MCH) related services, which left only one third of their time for discharging other programmatic activities. A time motion study from Wardha, Maharashtra, by Bhombe et al. [19] reported lack of clarity of job responsibilities among ANMs and non-effective utilization of available working time, which were quite bothersome. Thus, repeated sensitization regarding job responsibilities and effective time management among FHWs are needed. Lengthy tools emerged as another barrier in implementation. Tool pre-administration counseling regarding the importance of NCD screening, visiting houses on relatively free hours (i.e., afternoon) might be the possible strategies to overcome these problems.

Unawareness of community dwellers and deficiency in skill and knowledge of the FHWs emerged as key barriers of the program implementation. To alleviate this problem, repeated training need assessment and refreshment training of the FHWs are warranted. In this study, the most essential pieces of information needed to conduct NCD screening related activities were provided in the training module. This module may also be used for recapitulating important concepts and also might be utilized as a counseling aid for the community dwellers. A gender barrier in measurement of WC of males emerged as a challenge in CVD screening. The possible solutions could be seeking help from the other family members, community male volunteers, etc. The lack of availability of the logistics for NCD screening and late credit of honorarium for screening emerged as predominant barriers of the program implementation. For successful implementation of the program, sufficient and uninterrupted logistic supplies in terms of people, money and materials should be ensured.

### 4.1. Limitations of the study

First, the sample selection method used in this study was convenience sampling, considering feasibility and operational constraints. The selection and number of FHWs for undergoing training were decided by program administrators of the concerned districts. Thus, the findings of the research must be generalized with caution. Second, only the CVD component of the screening was prioritized in this research. The cancer screening component of the program will be addressed in separate future implementation research. Third, in few primary healthcare facilities, NCD portal skill of the ANMs could not be evaluated owing to non-availability of a functional tablet. Lastly, although as per protocol the SS visits were planned after three months of the training of FHWs, during implementation this could not be done due to the ongoing COVID-19 pandemic in the study area.

## 5. Conclusions

One day training on NCDs for FHWs was quite effective. However, for translating all the desired skills for CVD screening into action, periodic training needs assessment, and refreshment training and SS of FHWs might be fruitful strategies. In addition, timely uninterrupted logistic supplies for conducting screening related activities should also be ensured for quality assured delivery of services.
