# Supplementary material for: Capacity building among frontline health workers (FHWs) in screening for cardiovascular diseases (CVDs): Findings of an implementation study from Bihar, India
Source: AIMS Public Health. 2023 Mar 30;10(1):219–34. doi: 10.3934/publichealth.2023017 (PMC10091123; doi:10.3934/publichealth.2023017)
Supplement: Supplementary file 1 [file publichealth-10-01-017-s001.pdf]

Public Health

AIMS Public Health, 10(1): 219–234.
DOI: 10.3934/publichealth.2023017
Received: 20 November 2022
Revised: 8 March 2023
Accepted: 21 March 2023
Published: 30 March 2023

http://www.aimspress.com/journal/aimsph

*Research article*

# Capacity building among frontline health workers (FHWs) in screening for cardiovascular diseases (CVDs): Findings of an implementation study from Bihar, India

**Neeraj Agarwal[1,†], CM Singh[2,†], Bijaya Nanda Naik[2], Abhisek Mishra[3], Shamshad Ahmad[2], Pallavi Lohani[4], Saket Shekhar[5] and Bijit Biswas[6,*]**

[1] Community & Family Medicine, AIIMS, Bibinagar, Telangana, India
[2] Community & Family Medicine, AIIMS, Patna, Bihar, India
[3] Community Medicine & Family Medicine, AIIMS, Bhubaneswar, Odisha, India
[4] Community Medicine, Madhubani Medical College & Hospital, Keshopur, Bihar, India
[5] Community Medicine, Rama Medical College Hospital and Research Centre, Kanpur, U.P., India
[6] Community & Family Medicine, AIIMS, Deoghar, Jharkhand, India

* **Correspondence:** Email: drbijitbiswas@gmail.com.

† These two authors contributed equally.

## Supplementary I. Checklists for various skill ascertainment of FHWs.

Checklist for availability of logistics for Community Based NCD screening.

| | | |
|---|---|---|
| Family folder form | Yes [ ] | No [ ] |
| CBAC form | Yes [ ] | No [ ] |
| Non-stretchable measuring tape | Yes [ ] | No [ ] |
| Job-aids for counselling | Yes [ ] | No [ ] |

Checklist for 1st home visit.

| | | |
|---|---|---|
| Filled all the details of all family members in the family folder: | Yes [ ] | No [ ] |
| Filled CBAC forms for all persons aged ≥30years: | Yes [ ] | No [ ] |
| Informed regarding importance of NCDs: | Yes [ ] | No [ ] |
| Informed regarding importance of NCD screening: | Yes [ ] | No [ ] |
| Informed regarding modifiable risk factors of NCD: | Yes [ ] | No [ ] |
| Informed regarding non-modifiable risk factors of NCD: | Yes [ ] | No [ ] |
| Used job-aids (i.e., module) during counselling: | Yes [ ] | No [ ] |
| Able to calculate risk score of participants from CBAC form: | Yes [ ] | No [ ] |
| Referred all the persons aged ≥30years for NCD screening: | Yes [ ] | No [ ] |
| Prioritised high-risk persons in NCD screening: | Yes [ ] | No [ ] |

Checklist for follow-up home visit.

| | | |
|---|---|---|
| Checked drug compliance of NCD diagnosed patients: | Yes [ ] | No [ ] |
| Counselled regarding importance of drug compliance: | Yes [ ] | No [ ] |
| Explained importance of follow-up visit to doctor: | Yes [ ] | No [ ] |
| Motivated for 150 minutes physical activity or yoga per week: | Yes [ ] | No [ ] |
| Motivated for salt restriction in diet: | Yes [ ] | No [ ] |
| Motivated for oil restriction in diet: | Yes [ ] | No [ ] |
| Used job-aids (i.e., module) during counselling: | Yes [ ] | No [ ] |

Checklist for logistics availability for weekly NCD clinics.

| | | |
|---|---|---|
| Weighing machine | Yes [ ] | No [ ] |
| Height measurement tool/ stadiometer | Yes [ ] | No [ ] |
| Non-stretchable measuring tape | Yes [ ] | No [ ] |
| Blood pressure machine | Yes [ ] | No [ ] |
| Glucometer machine | Yes [ ] | No [ ] |
| Glucometer strips | Yes [ ] | No [ ] |
| Lancet/ needle | Yes [ ] | No [ ] |
| Cotton/ alcohol swab | Yes [ ] | No [ ] |
| Gloves/ Handwashing facility | Yes [ ] | No [ ] |
| Tablet for data entry on NCD portal | Yes [ ] | No [ ] |
| Internet connection for data syncing | Yes [ ] | No [ ] |

Checklist for NCD portal skill.

| | | |
|---|---|---|
| Able to open NCD portal | Yes [ ] | No [ ] |
| Able to log in on NCD portal | Yes [ ] | No [ ] |
| Able to enter family folder in NCD portal | Yes [ ] | No [ ] |
| Able to enter CBAC form in NCD portal | Yes [ ] | No [ ] |
| Able to sync data in NCD portal | Yes [ ] | No [ ] |
| Able to retrieve data in NCD portal | Yes [ ] | No [ ] |
| Able to check due-list in NCD portal | Yes [ ] | No [ ] |

Checklist for weight measurement.

| | | |
|---|---|---|
| 1. Weighing scale kept at a flat surface: | Yes [ ] | No [ ] |
| 2. Calibrated the weighing scale: | Yes [ ] | No [ ] |
| 3. Asked participant to remove heavy objects like shoes, winter clothing's, head gear | Yes [ ] | No [ ] |
| 4. Asked participant to stand at centre of the scale with slight gap between the feet: | Yes [ ] | No [ ] |
| 5. Asked participant too look straight: | Yes [ ] | No [ ] |
| 6. Looked at the reading from above: | Yes [ ] | No [ ] |

Checklist for height measurement.

| | | |
|---|---|---|
| 1. Made the participant stand on a flat surface | Yes [ ] | No [ ] |
| 2. Made the participant stand against the stadiometer or wall | Yes [ ] | No [ ] |
| 3. Asked participants to remove headgear and shoes: | Yes [ ] | No [ ] |
| 4. Correctly positioned the participant with 4 points touching the wall or stadiometer: (back of head, shoulder, lower back, ankle) | Yes [ ] | No [ ] |
| 5. Asked participant to look straight with slight gap between the feet: | Yes [ ] | No [ ] |
| 6. Kept a cardboard or hardboard register above head of the participant: | Yes [ ] | No [ ] |
| 7. Looked at the reading from above: | Yes [ ] | No [ ] |

Checklist for waist circumference measurement.

| | | |
|---|---|---|
| 1. Used non-stretchable measuring tape: | Yes [ ] | No [ ] |
| 2. Asked participant to stand and look straight with slight gap between two feet: | Yes [ ] | No [ ] |
| 3. Asked participant to reveal abdominal part or remove heavy clothing: | Yes [ ] | No [ ] |
| 4. Put the measuring tape at the level of umbilicus: | Yes [ ] | No [ ] |
| 5. Checked for fit (not too tight, not too loose): | Yes [ ] | No [ ] |
| 6. Checked alignment of the tape all around the participant: | Yes [ ] | No [ ] |
| 7. Measured waist circumference at the end of normal exhalation: | Yes [ ] | No [ ] |

Checklist for blood pressure measurement.

| | | |
|---|---|---|
| 1. Asked the participant whether they have done following in past 30 minutes: | | |
| 1.1. Consumed Tea/Coffee | Yes [ ] | No [ ] |
| 1.2. Smoked | Yes [ ] | No [ ] |
| 1.3. Heavy exercise | Yes [ ] | No [ ] |
| 1.4. Consumed alcohol | Yes [ ] | No [ ] |
| 2. Asked whether the participant need to empty the bladder | Yes [ ] | No [ ] |

3. Position of the participant while measuring:
    3.1. Seated Yes [ ] No [ ]
    3.2. Back supported Yes [ ] No [ ]
    3.3. Feet resting firmly on the floor Yes [ ] No [ ]
    3.4. Feet not crossed Yes [ ] No [ ]
4. Position of the arm
    4.1. Supported from elbow till palm Yes [ ] No [ ]
    4.2. At the level of the heart Yes [ ] No [ ]
    4.3. Palm facing up Yes [ ] No [ ]
5. Placement of the cuff:
    5.1. 2–3 cm/ 2 finger above the antecubital fossa Yes [ ] No [ ]
    5.2. Covering brachial artery Yes [ ] No [ ]
    5.3. Edge of the cuff is not restricted by clothing Yes [ ] No [ ]
    5.4. Checked for fit (not too tight, not too loose) Yes [ ] No [ ]
6. Documentation of reading:
7. Asked the participant not to move Yes [ ] No [ ]
8. Asked the participant not to talk Yes [ ] No [ ]
9. Clicked the START/STOP button Yes [ ] No [ ]
10. Checked and documented reading after 30–45 seconds Yes [ ] No [ ]

Checklist of random blood sugar measurement.

1. Explained the procedure to the participant: Yes [ ] No [ ]
2. Checked availability of lancet, test stripes and functional glucometer Yes [ ] No [ ]
3. Put on gloves/ washed hands prior to testing: Yes [ ] No [ ]
4. Cleaned the participants finger to be pricked with cotton: Yes [ ] No [ ]
5. Insert the test strip in glucometer: Yes [ ] No [ ]
6. Hold and pricked the finger of the participant with lancet/ needle: Yes [ ] No [ ]
7. Confirmed that the machine is ready for the test: Yes [ ] No [ ]
8. Discarded the first drop then applied the second drop of blood to the test strip: Yes [ ] No [ ]
9. Asked participant to lightly press pricked finger with cotton: Yes [ ] No [ ]
10. Recorded and documented the test result: Yes [ ] No [ ]
11. Properly disposed generated wastes Yes [ ] No [ ]

AIMS Press

© 2023 the Author(s), licensee AIMS Press. This is an open access article distributed under the terms of the Creative Commons Attribution License (http://creativecommons.org/licenses/by/4.0).
